# Triblock Copolymer Toughening of a Carbon Fibre-Reinforced Epoxy Composite for Bonded Repair

**DOI:** 10.3390/polym10080888

**Published:** 2018-08-08

**Authors:** Andrew D.M. Charles, Andrew N. Rider

**Affiliations:** Aerospace Division, Defence Science and Technology Group, Fishermans Bend 3207, Australia; andrew.charles@dst.defence.gov.au

**Keywords:** carbon fibre, nano-structures, mechanical properties, polymer-matrix composites

## Abstract

Epoxy resins are the most widely used systems for structural composite applications; however, they lack fracture toughness, impact strength and peel strength due to high cross-linking densities. Use of conventional toughening agents to combat this can lead to reductions in mechanical, thermal and processability properties desirable for bonded composite applications. In this work, an asymmetric triblock copolymer of poly(styrene)*–b–*poly(butadiene)*–b–*poly(methylmethacrylate) was used to modify an epoxy resin system, with the materials processed using both vacuum bag and positive pressure curing techniques. Interlaminar fracture toughness testing showed improvements in initiation fracture toughness of up to 88%, accompanied by a 6 °C increase in glass transition temperature and manageable reductions in gel-time. Shear testing resulted in a 121% increase in ultimate shear strain with only an 8% reduction in shear strength. Performance improvements were attributed to nano-structuring within the toughened resin system, giving rise to matrix cavitation and dissipation of crack front strain energy upon loading.

## 1. Introduction

Epoxy resins are the most widely used thermoset systems for structural applications, due to their excellent chemical and mechanical properties, low shrinkage, good compatibility with a range of different reinforcing fibres [1] and relatively high glass transition temperatures (*T_g_*). This latter property results from the highly cross-linked nature of the polymer, which unfortunately also translates into poor fracture toughness, poor resistance to crack propagation and low impact and peel strengths. Extensive research has been conducted over the past few decades with the aim to improve the toughness of epoxy resins, predominantly through the incorporation of modifiers or particulates [2].

A range of different modifiers have featured throughout the literature, including elastomers [3,4], thermoplastics [5,6] and nanoparticle modifiers [7,8,9,10]. Most of the modifiers used to toughen epoxy resin systems are of an elastomeric or thermoplastic nature, with the use of reactive diluents and inorganic/hybrid modifiers also featured throughout literature.

The use of elastomers such as liquid rubbers provide significant improvements to the fracture toughness of epoxy systems; however, this can lead to reductions in *T_g_* and modulus, as well as undesirable mechanical and chemical performance at elevated temperatures [4]. Furthermore, the benefits of elastomeric modifiers decrease as the cross-linking density of the epoxy resin system increases [2]. In contrast to this, the use of rigid thermoplastic particles as toughening modifiers can lead to improvements in epoxy fracture toughness without sacrificing the thermal properties and strength of the system [5]. This is because the thermoplastic particles are tough, ductile, chemically and thermally stable and have relatively high *T_g_’s* compared to elastomeric modifiers. Despite the advantages, the toughness improvements achieved through thermoplastic modifiers are generally poor when compared with elastomeric modifiers [2].

Particle sizes for elastomeric or thermoplastic modifiers are typically in the order of 1–5 μm in diameter at resin volume fractions of between 5–20 wt %. These particle sizes and loadings can lead to substantial increases in uncured resin viscosity, reducing processability, which can be problematic, particularly for liquid moulding techniques. Furthermore, these relatively large particle sizes prohibit the use of such systems with infusion processes, where the modifiers are often filtered from the resin by reinforcing fabric material [10,11].

In contrast to elastomers and thermoplastic modifiers, nanophase modifiers have been shown to provide improvements in modulus, strength and fracture toughness. This has been demonstrated with carbon black and nano-clay [7], nano-silica [8] and block copolymers [9,12,13,14,15]. Block copolymers are particularly unique, as their toughening properties arise from the formation of unique nanostructures within the epoxy matrix, which provide improvements in toughness with minimal impact on *T_g_* and modulus. The formation of these nanostructures is a more efficient toughening mechanism, with lower volume fractions required for equivalent toughening when compared to other particle types. The ability to dissolve such copolymers with a host monomer is also advantageous, providing consistent dispersion. The small size of the nanostructures formed also allows for their use in composites with small inter-fibre spacing, thin bond-line adhesives and in resin transfer moulding (RTM) applications where other particles may be filtered by the composite fibres during the infusion process [16].

A number of studies have focused on the effect block copolymers have on the mechanical properties and fracture performance of carbon fibre-reinforced polymer (CFRP) composite systems. For example, Chong et al. [17] demonstrated improvements in fracture toughness for increasing block copolymer content in single-edge notch bending (SENB) for CFRP structures manufactured using a resin infusion using the flexible tooling (RIFT) method. Vacuum assisted resin transfer moulding (VA-RTM) was used in [18] to enhance the toughness of a high-performance RTM6 matrix system (Hexcel, Stamford, CT, USA) with interlaminar fracture toughness explored using the double cantilever beam coupon design. A hot press method was also used to cure pre-impregnated material manufactured using an acrylic block copolymer, with mode I and mode II fracture toughness, open hole compression, tension and impact testing performed to characterise resulting mechanical performance [19]. Kamar et al. [20] explored the differing toughening mechanisms and mechanical performance of epoxy resins toughened with carboxyl-terminated butadiene-acrylonitrile (CTBN) and a poly(styrene)–*b*–poly(butadiene)–*b*–poly(methylmethacrylate) (SBM) triblock copolymer, with fracture toughness evaluated through the use of compact tension (CT) coupons. Further work examined the effect of fibre sizing and block copolymer content effects for autoclave-cured CFRP [21].

To date, the use of block copolymer-toughened epoxy resins in a composite wet lay-up and vacuum bag cure scenario has yet to be explored. The vacuum cure approach is typical of bonded composite repair processes, with successful repair implementation critically relying on knowledge of resin handleability and the cured resin properties, particularly the bonding resin’s shear performance. To the best of the authors’ knowledge, shear stress–strain performance has yet to be reported for block copolymer toughened epoxy resin. Thus, this work focuses on assessing the shear, interlaminar fracture toughness and flexural properties of a block copolymer modified epoxy resin system, with coupons manufactured using a wet lay-up/vacuum bag cure method and typical composite bonded repair laminating epoxy resin. Comparisons are made to autoclave-cured material, with resin gel time, viscosity and thermomechanical performance also being assessed.

## 2. Materials and Methods

### 2.1. Materials

The resin system utilised was a 2-part bisphenol A/F epoxy known as K3600, provided by Huntsman (Huntsman Performance Products, Brooklyn, Australia). For the block copolymer a triblock of polystyrene (PS block), 1,4-polybutadiene (PB block) and syndiotactic poly(Methyl methacrylate) (PMMA block), also known as SBM, was provided by Arkema (Colombes, France) as part of their Nanostrength^®^ range, with E21 grade used in the work. The number average molar mass and wt % composition of each of the blocks in the copolymer were [13] PS: 27,000 g/mol and 22 wt %, PB: 11,000 g/mol and 9 wt % and PMMA: 84,000 g/mol and 69 wt %.

To incorporate the triblock copolymer into the resin, the SBM powder was firstly dried at 110 °C for 3 h in a laboratory drying oven and then ground using a mortar and pestle. The copolymer was then added to the epoxy resin at a 10 wt % (to final resin weight) and dispersed using a laboratory mixer and low torque mixing head at room temperature. Heat was then applied to the mixture via a temperature-controlled hot plate and stirred at 80 °C until complete dissolution was observed, usually after 3–4 h. This mixture was then combined with the resin hardener at 100:30 parts-by-weight active resin (excluding SBM) to hardener.

The resin was used neat (with no SBM) and toughened in combination with two different carbon fibre reinforcements: (i) a unidirectional IM7 3k carbon known as Hex Tow IM7 (Hexcel, Stamford, CT, USA) and (ii) a 3k carbon plain-weave fabric, 195 g/m^2^, known as RC200P (Gurit, Zurich, Switzerland).

### 2.2. Coupon Manufacture

**Figure 1:**
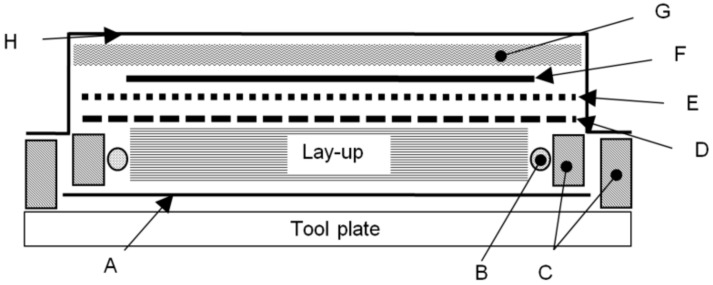
Coupon bagging procedure. A: 25 µm release film, B: carbon fibre tow, C: rubber vacuum tape, D: perforated 25 µm release film, E: polyester peel-ply, F: aluminium caul plate, G: breather cloth and H: nylon bagging film.

Four point bend flexure coupons were manufactured with 16 plies of the plain weave fabric, while 16 plies of the uni-directional fabric was used to manufacture double cantilever beam (DCB) coupons. For the DCB coupons, a 12.5 µm thick polytetrafluoroethylene (PTFE) film was used at the mid-plane of the coupon to form an initiation site for the delamination. Both coupon sets were prepared using a wet lay-up procedure, bagged using the technique depicted in Figure 1 and left to debulk for at least 8 h under 75 ± 5 kPa vacuum pressure. The coupons were then either cured under vacuum in a laboratory drying oven under 75 ± 5 kPa vacuum pressure or in an autoclave under 586 kPa positive pressure. Cure was achieved in both cases using the resin manufacturer cure cycle: 24 h at 25 °C, followed by a 3 h post-cure at 80 °C. Coupons were then machined to size using a water lubricated pneumatic diamond saw.

Thick Adherend Lap Shear (TALS, ASTM D5656 [22]) coupons were also manufactured for testing using 9.5 mm thick 2024-T3 aluminium alloy plates. To prepare the plates for bonding, the surfaces were solvent degreased with methyl ethyl ketone (MEK), abraded with Scotch-Brite^TM^ 7447 (3M, St. Paul, MN, USA) hand pads and left to dry in an air-circulating oven for 20 min at 80 °C. The surfaces were then grit blasted with 50 μm aluminium oxide grit using dry nitrogen gas as a propellant at a pressure of approximately 450 kPa. The plates were then submerged in an aqueous solution of 1% γ-glycidoxypropyl trimethoxy silane (γ-GPS) to 99% distilled water for 10 min and then dried for one hour at 110 °C in a laboratory drying oven. A 130 µm nylon scrim was used to maintain a consistent bondline and the plates cured at 25 °C and 38% relative humidity in an environmental conditioning chamber for 24 h, with positive pressure of 75 kPa applied through use of dead weights. A post-cure was then performed in a laboratory drying oven at 80 °C for 3 h. Once cured, the 25.4 mm wide coupons were cut using a vertical turret mill.

For Dynamic Mechanical Thermal Analysis (DMTA), neat and SBM-toughened resin were cured onto a flat PTFE film bordered with Flashbreaker^®^ tape (Airtech International, Huntington Beach, CA, USA) coated cork dams. Once cured, the resin block was machined using a computer numerically controlled router for testing.

### 2.3. Flexural Testing

Four point bend flexure testing (4PBT) was used to characterise the flexural strength (*F_4PB_*) and tangent flexural modulus in bending (*E_B_*) in accordance with ASTM D6272 [23]. The flexural strength was defined as the maximum stress in the outer fibre of the specimen at the moment of break and was determined using Equation (1):

(1) $$S=\frac{PL}{bd^{2}}$$

where *S* was the fibre stress, *P* the load applied at break, *b* the specimen width, *d* the specimen thickness and *L* the support span. For all tests, *L* was set equal to sixteen times the specimen thickness and the loading span set equal to one third of the support span. To avoid premature failure due to localised stress directly under the loading noses of the fixture, 6.2 mm loading noses were used for the test. *E_B_* was determined using Equation (2):

(2) $$E_{B}=0.21L^{3}\frac{m}{bd^{3}}$$

In this equation, *m* was calculated as the slope of a tangent drawn to the steepest initial straight-line portion of the applied load to mid-span deflection curve. Mid-span deflection was measured using a linear variable differential transformer (LVDT) placed on the top surface of each specimen and connected to a WE7000 data acquisition unit (Yokogawa, Tokyo, Japan), sampling at 10 Hz. The loading rate for each coupon set was based on the average coupon thickness, test support span and an outer fibre strain rate of 0.01 mm/mm per guidance provided in ASTM D6272.

### 2.4. Mode-I Interlaminar Fracture Toughness

**Figure 2:**
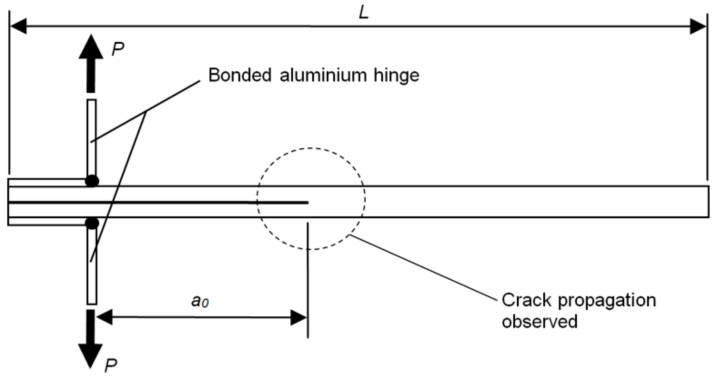
Double cantilever beam (DCB) specimen and loading configuration.

For the double cantilever beam (DCB) testing (per ASTM D5528 [24]), load was applied by way of aluminium piano hinges bonded using EA9309.3NA past adhesive (Henkel, Düsseldorf, Germany) as shown in Figure 2, with the crack propagation, *a*, recorded using a travelling microscope. The mode I interlaminar fracture toughness (*G_Ic_*) was determined via Modified Beam Theory using Equation (3):

(3) $$G_{Ic}=\frac{3P\delta }{2b\left(a+\Delta \right)}$$

where *P* was the applied load, *δ* the test machine cross-head displacement and *b* the specimen width. *G_Ic_* vs. crack length curves were developed for all coupons tested, and Equation (3) applied at the point of visual crack indication (VIS), the point of deviation from linearity of the load-displacement curve (NL) and at the maximum load point (MAX) of each coupon. Generally, the NL point is considered the most representative, as it does not rely on a visual indication for the crack growth, which may occur within the sample interior [25]. Furthermore, fibre bridging after crack initiation and growth can contribute to the measured *G_Ic_*, and will thus artificially enhance the calculated MAX *G_Ic_*.

**Figure 3:**
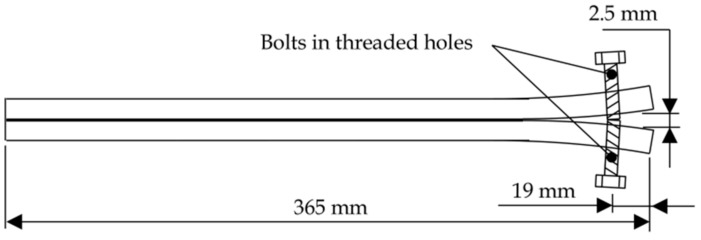
Schematic of long crack extension (LCE) specimen.

To compare the interlaminar fracture toughness values determined via the DCB method with bulk matrix fracture behaviour, long crack extension (LCE) was also performed in accordance with a Lockheed Martin standard derived from ASTM standard D3433 [26], D3762 [27] and Boeing specification BSS 7208 [28]. The coupons for this test comprised of 12.75 mm thick, 25 mm wide 2024-T3 aluminium alloy plates with pre-drilled and tapped holes centrally positioned 19 mm from one end as shown in Figure 3. The surfaces of these plates were prepared using the same procedures applied to the TALS coupons and then bonded together with the adhesive of interest, with bondline thickness maintained through the use of 200 µm copper shim. Flashbreaker^®^ 1R tape (75 µm thick) was used to introduce a pre-crack region covering 25 mm aft of the bolt. Cure was performed within a laboratory drying oven at 25 °C for 24 h, with positive pressure of 75 kPa applied through use of dead weights. A post-cure was then performed in a laboratory drying oven at 80 °C for 3 h.

Once cured, an opening displacement of 2.5 mm was applied by screwing the bolts shown in Figure 3 and the crack length measured using an optical travelling microscope at 0, 25 and 49 h intervals. Using the crack opening displacement, *Y*, and crack length, *a*, resin fracture toughness was determined via Equation (4),

(4) $$G_{Ic}=\frac{Y^{2}Eh^{2}\left\{3\left[{\left(a+0.6h\right)}^{2}+h^{2}\right]\right\}}{16{\left[{\left(a+0.6h\right)}^{3}+ah^{2}\right]}^{2}}$$

where *h* and *E* are the adherend thickness and adherend Young’s modulus respectively.

### 2.5. Shear Testing

For TALS testing, two extensometers attached either side of the bond region of the specimen, and also connected to a Yokogawa WE7000 data acquisition unit, were used to monitor deflection during specimen loading. Testing was performed using an Instron 1185 (Instron, Norwood, MA, USA) electromechanical universal testing machine with a 100 kN load cell and a loading rate of 2455 N/min. This same test machine was used to test both DCB and 4PBT coupons.

### 2.6. Thermomechanical Testing

Dynamic Mechanical Thermal Analysis (DMTA) testing was performed using a Polymer Laboratories Mark III dynamic mechanical thermal analyser. All specimens were loaded in a single cantilever bending mode (flexure) with 1, 10 and 15 Hz oscillatory frequencies applied. The specimen temperature was ramped from 40 °C to a maximum of 200 °C at a ramp rate of 3 °C/min. Material loss and storage moduli (E” and E’) were recorded during testing, with three consecutive runs performed on each sample to provide an indication of the degree of cure and the associated shift in mechanical response. The *T_g_* was defined as the temperature corresponding to the peak in the tan(*δ*) response (equal to E”/E’), where *δ* was considered the phase angle shift between stress and strain vectors.

To gauge the impact of the SBM nanophase on resin workability, the resin gel time at different ambient temperatures was investigated using a Gelnorm-TC gel timer (Gel Instrumente AG, Oberuzwil, Switzerland), with the mixed resin sample temperature controlled through use of a thermostatic bath. Resin cure temperatures of 20 °C, 30 °C and 40 °C were investigated. In addition, viscosity of the K3600 system with and without the copolymer (at 10 wt % loading) was quantified using a Brookfield DV-II digital rotational viscometer (AMETEK Brookfield, Middleboro, MA, USA) and a temperature-controlled sample vial connected to a Brookfield TC502 circulating water bath. Viscosity measurements were performed from 15 °C to 35 °C in 5 °C increments.

### 2.7. Fractography

Two different specimen types were prepared for fractographic analysis. The first were excised from the crack tip of each of the DCB specimens using a water lubricated diamond saw. These coupons, approximately 10 × 10 mm square, were separated along the fracture plane using a scalpel to propagate the interlaminar crack to complete coupon failure and the surface mounted onto a scanning electron microscope stub using epoxy resin and grounded to the stub with tabs of aluminium tape. These coupons were then sputter coated with 2 nm of iridium and interrogated using a LEO 1530VP Field Emission Scanning Electron Microscope (FESEM) (LEO Electron Microscopy, Thornwood, NY, USA). Additional fracture surfaces were also produced from neat and SBM-toughened K3600 resin coupons by liquid nitrogen cooling and brittle fracture. After fracture, redundant material was removed using a micro-saw and the specimens again sputter coated with 2 nm of iridium and interrogated using the FESEM.

## 3. Results and Discussion

### 3.1. Gel Time and Viscosity

**Figure 4:**
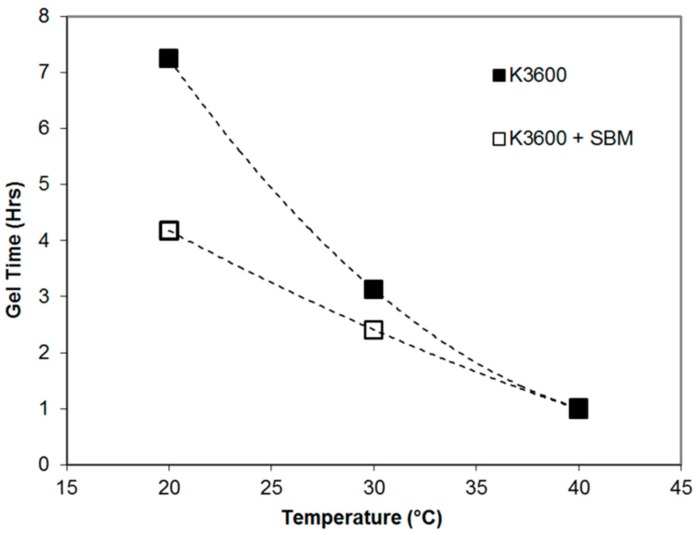
Gel time results as a function of cure temperature for neat and toughened resin systems.

The gel time results for the neat and toughened systems are shown in Figure 4, showing a quadratic trend in gel time reduction for increasing resin temperature. Both systems reached the same gel time at 40 °C, suggesting that the addition of the SBM did not impact on the cure kinematics, but rather the viscosity of the resin. From the viscosity measurements, viscosity was found to increase with the addition of the copolymer, and decrease with increasing temperature as shown in the results of Table 1, with a maximum of 16 Pa·s found for the K3600 + E21 at 15 °C.

Chong and Taylor [17] previously demonstrated an increase in viscosity for a standard diglycidyl ether epoxy resin and 10 wt % Arkema E21 SBM (Colombes, France) through use of a rheometer, increasing from ~0.5 Pa·s to ~40 Pa·s with the addition of the SBM at 35 °C. Despite the increase in viscosity observed, the handleability of the resin was sufficient to permit manufacture of all test coupons using the standard wet-lay-up and vacuum bag methodology detailed previously, all of which were performed under laboratory ambient conditions, nominally 23 °C and 70% relative humidity. Based on this temperature, the gel time of the SBM-toughened resin system would be approximately 3.6 h. This time is more than sufficient to perform the necessary composite wet lay-up and repair application procedures typical of small to medium sized (up to approximately 400 mm in diameter) stepped or scarfed wet lay-up repairs of up to 8 plies to flat composite components.

### 3.2. Dynamic Mechanical Thermal Analysis

**Figure 5:**
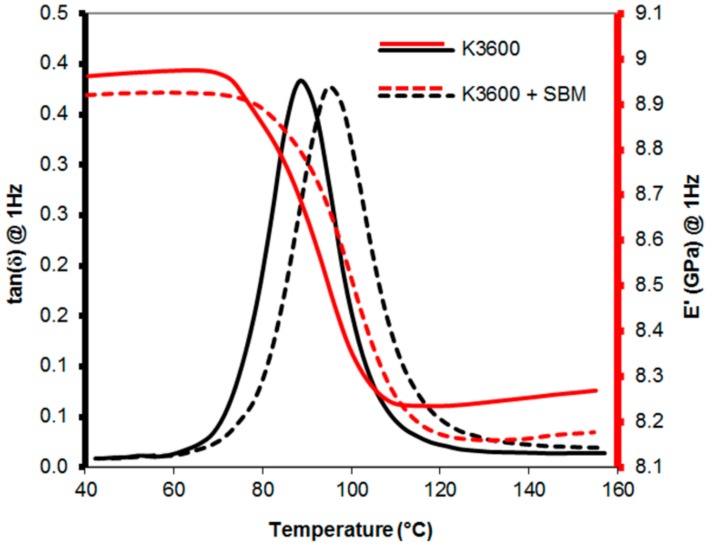
Dynamic Mechanical Thermal Analysis (DMTA) curves for the neat and poly(styrene)*–b–*poly(butadiene)*–b–*poly(methylmethacrylate) (SBM) toughened K3600 resins, averaged over the three runs performed at 1 Hz.

The tan(*δ*) and E’ responses of neat and SBM-toughened resins measured at 1 Hz are shown in Figure 5, averaged over the three runs performed. The results show a single peak in the tan(*δ*), indicating a single material phase, with a shift in peak with the addition of the SBM showing an increase in resin *T_g_* from 88 ± 6 °C to 95 ± 5 °C when SBM was added. As the *T_g_* is driven by the properties of the cross-linked epoxy matrix, no change or increase in the *T_g_* would suggest complete phase separation of the SBM occurred. This was also supported by the visual appearance of the K3600 + SBM coupons manufactured (with no fibres), which were transparent to visible light during SBM introduction and mixing but opaque upon cure. This would suggest that the SBM is initially miscible in the host epoxy matrix during mixing, but separates during cure to form light scattering structures, resulting in an opaque solid material. Other authors have shown that the presence of the soft, flexible PB block in the copolymer can lead to a reduction in the storage modulus with increasing temperature prior to the main transition [20].

**Figure 6:**
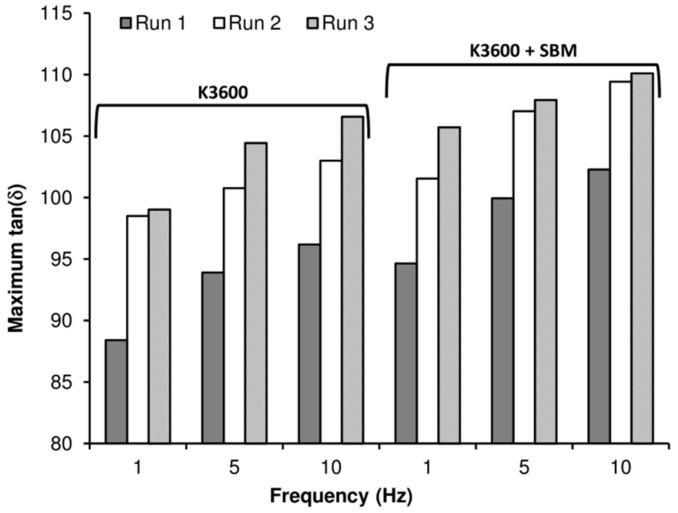
Peak tan(*δ*) response as a function of applied frequency and number of runs for the neat and SBM-toughened K3600 resin coupons analysed via DMTA.

To explore the effects of heating of the sample as a result of the DMTA testing, three consecutive runs were performed for each coupon tested, with the results for the three frequencies investigated shown in Figure 6. Comparing the *T_g_* (measured as the peak in tan(*δ*) response) for the three applied frequencies, this increased with increasing applied frequency. As the *T_g_* is essentially a measure of the molecular relaxation in the material, which is in-turn dependent upon the material temperature, high test frequencies result in molecular relaxations occurring at higher temperatures, resulting in a measured increase in *T_g_*.

Within each frequency measured, we also see an increase in *T_g_* between consecutive runs of the same sample. This suggests that the heating applied during DMTA analysis (up to 200 °C) is leading to a progressive post-curing of the sample. As *T_g_* can be empirically related to the cross-linking density within the polymer [29], this would suggest that the additional heating is leading to an increase in cross-linking density, which has been shown to impact the effectiveness of toughening agents in elastomer-modified epoxies [30].

### 3.3. Mode I Interlaminar Fracture Toughness

**Figure 7:**
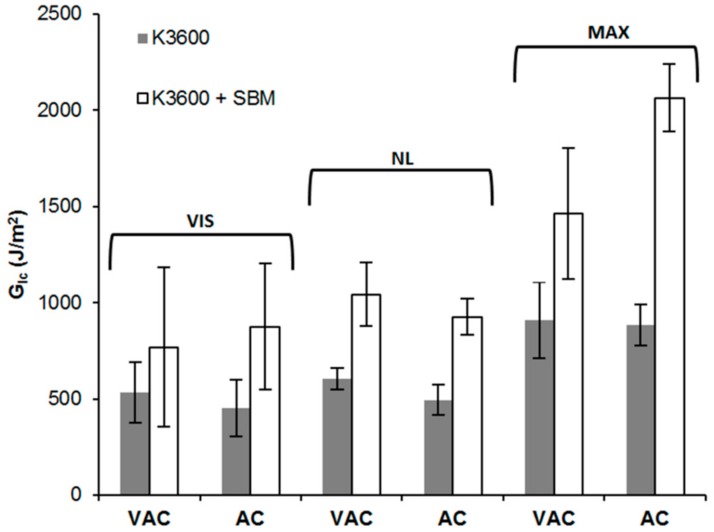
Visual (VIS), Non-linear (NL) and maximum (MAX) *G_Ic_* results for vacuum bag (VAC) and autoclave (AC) cured neat and SBM-toughened K3600. Error bars show standard deviation in each data set.

**Figure 8:**
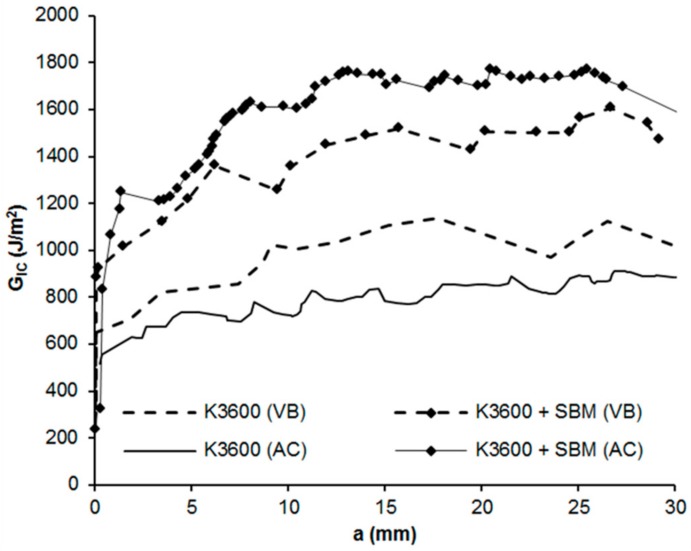
Representative delamination resistance curves (R-curves) for each of the coupon sets investigated. VAC: Vacuum bag cure method, AC: Autoclave cure method.

From the DCB testing, all coupons tested failed via crack propagation from the introduced PTFE insert as expected, with VIS, NL and MAX indications for *G_Ic_* presented in Figure 7 and representative delamination resistance curves (R-curves) shown in Figure 8. As can be seen from these results, in all cases, interlaminar *G_Ic_* increases with increasing crack increment and stabilises after approximately 10–15 mm of crack growth. The principal cause of the increasing *G_Ic_* arises from the formation of crack bridging fibres within the coupon, resulting from crack front switching between one fibre-matrix interface to an adjacent fibre-matrix interface, giving rise to bridging fibres. These bridging fibres provide traction forces which increase the observed toughness during testing as identified in the three different measurement regimes (VIS, NL and MAX).

Typically, in structural composite applications, delaminations form between plies of dissimilar orientation, limiting the likelihood of fibre bridging. For this reason, VIS and NL *G_Ic_* values are considered more representative as they provide an initiation value of the *G_Ic_* with limited contribution from the bridging fibres of the specimen. Of these, the VIS indication provides a high standard deviation error in the results, which is likely due to the formation and growth of un-observed delaminations internal to some of the coupons in this set, skewing the average result and leading to higher errors. In contrast, the NL indicator provided the lowest error, with average improvements observed in both cure methods with the addition of the SBM, 73% for the vacuum bag cure and 88% for the autoclave cure.

These results compare well with other reported SBM-modified epoxy results in the literature, where DCB testing of an SBM modified epoxy led to propagation interlaminar *G_Ic_* improvements with increasing concentrations of SBM within a host epoxy of approximately 60% for 5 wt % SBM and 115% for 10 wt % SBM [21]. Improvement in DCB propagation interlaminar *G_Ic_* for an SBM-modified epoxy was found to be greatest at 2.5 wt %, approximately 42%, and reduced for higher concentrations of SBM, 30% improvement for 5 wt %, 24% for 7.5 wt % and only 3% for 10 wt % concentration of SBM [17]. This reduction in propagation interlaminar *G_Ic_* with increasing SBM content was hypothesised to be a result of improved fibre-matrix bonding at higher SBM loadings and an accompanied reduction in fibre bridging for these loadings. As highlighted before, this fibre bridging mechanism contributes to the observed propagation *G_Ic_* measured using the DCB technique.

From the LCE testing, a neat K3600 *G_Ic_* value of 329.71 J/m^2^ and K3600 + SBM *G_Ic_* value of 878.48 J/m^2^ was achieved after the crack was allowed to equilibrate after 49 h. In all cases, cohesive failure was observed within the bondline. Comparing these results with the DCB testing values, the K3600 and K3600 + SBM values determined by LCE are approximately 40% and 10% lower than the respective NL *G_Ic_* values determined by DCB. This difference highlights the contribution of bridging fibre traction forces which increase the observed toughness in the DCB test.

### 3.4. Shear Properties

**Figure 9:**
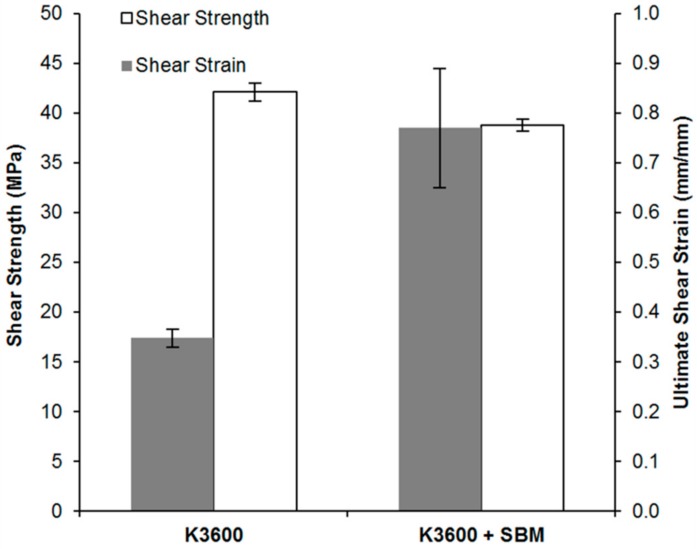
Shear strength and ultimate shear strain results for neat and SBM-toughened K3600. Error bars show standard deviation in each data set.

**Figure 10:**
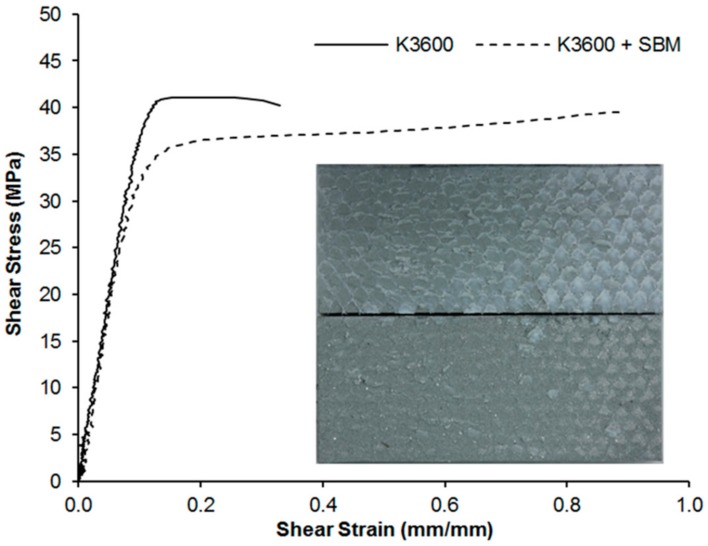
Representative shear stress–strain curves for the neat and toughened resin systems. The insert shows a 25.4 mm wide representative failure surface showing evidence of adhesive and bondline control scrim on both adherend surfaces.

The shear stress and ultimate shear strain results from TALS testing are shown in Figure 9, with representative shear stress–strain curves for the two systems shown in Figure 10. For all coupons tested, 5 TALS coupons from each of the neat K3600 and K3600 + SBM sets, cohesive failure occurred through the adhesive bondline, with an example failure surface shown in Figure 10 insert. As can be seen from Figure 9, an 8% reduction in the average shear strength occurred with the introduction of the SBM, accompanied by a 121% increase in the average ultimate shear strain. This increase in strain to failure is clearly seen by the increase in plastic zone in the stress–strain curves of Figure 10 and is directly attributable to the increase in matrix toughness as a result of the SBM addition. In the shear loading condition, as experienced within the TALS specimen, joint failure arises from the nucleation, growth and subsequent joining of cracks within the bondline [31]. Crack nucleation in the shear condition arises due to localised mode I fracture of the adhesive at a 45° direction relative to the shear load. As demonstrated previously, an increase in the mode I toughness is observed for the SBM-toughened system, which also translates to an increase in the strain to failure due to the crack nucleation mechanisms at play.

### 3.5. Flexural Performance

**Figure 11:**
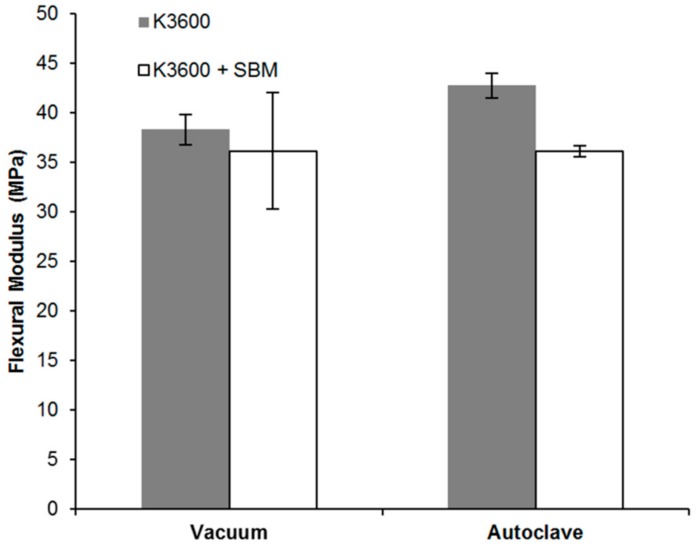
Flexural modulus (measured by four point bending) results of vacuum bag- and autoclave-cured neat and SBM-toughened K3600. Error bars show standard deviation in data set.

The flexural modulus of both vacuum bag- and autoclave-cured neat and SBM-toughened systems are shown in Figure 11. All coupons tested failed within the 5% outer fibre strain limit specified by the standard. As can be seen in Figure 11, a slight reduction in average flexural modulus was observed with the addition of the SBM, 5.7% for the vacuum bag-cured system and 15.5% for the autoclave-cured system. This reduction can be partly explained by a reduction in the fibre volume fraction (*v_f_*) for the SBM-toughened coupons, with matrix digestion (in accordance with ASTM D3171 [32]) of the vacuum cured panels indicating a neat K3600 coupon *v_f_* of 45.8 ± 0.4%, reducing to 43.4 ± 1.0% for the K3600 + SBM system. Based on these *v_f_* values, using a simple Rule of Mixtures (ROM) the predicted tensile modulus for neat and K3600 toughened systems were 32.2 ± 0.6 GPa and 30.7 ± 1.4 GPa respectively. This equates to a reduction in predicted tensile modulus of 4.7 ± 1.0%, which is in-line with the observed flexural modulus reduction of 5.7%. The *v_f_* of the autoclave-cured coupons was found to be 47.1 ± 1.0% for the neat K3600 coupons, decreasing to 42.4 ± 1.2% with the addition of the SBM, accounting for the respective changes in modulus for these coupon sets when compared to the vacuum bag-cured coupons.

The reduction in *v_f_* was symptomatic of an increase in thickness for all SBM coupons manufactured, with the vacuum bag-cured panels increasing by approximately 19% from 3.8 mm (neat K3600) to 4.6 mm (K3600 + SBM) and the autoclave-cured coupons increasing by approximately 22% from 2.1 mm to 2.6 mm. This increase in thickness is attributed to the increase in resin viscosity with the addition of the SBM as quantified previously. A similar trend in *v_f_* was observed by Kamar et al. [21], decreasing from 63.8 ± 1.0% to 56.2 ± 2.0% with 5 wt % SBM, resulting in statistically insignificant changes to the flexural strength or stiffness of the epoxy coupons studied.

The modulus results for the vacuum bag-cured system in Figure 11 show larger spreads in the standard deviation when compared to the autoclave-cured results. This is thought to be a result of higher porosity due to the vacuum applied during cure, with matrix digestion of these coupons indicating an average void content of 3.5 ± 0.3%, higher than the 1.0 ± 0.7% for the positive pressure autoclave-cured coupons. This is a common issue encountered for vacuum bag-cured components as the consolidation pressure applied is generally much lower than can be achieved via positive pressure autoclave cures. Generally, consolidation pressure and vacuum are used to reduce porosity from the composite prior to and during cure. For elevated-temperature cures, in order to prevent volatile gas expansion, the resin must be placed under hydrostatic pressure and this is achieved through positive pressure cures such as that available through an autoclave. For bonded composite repair where removal of a part of a larger component is often not possible, or the part is too large for an autoclave, vacuum bag methods have been shown to be a highly versatile repair method. This method can produce good quality repairs, particularly for lower temperature resin systems [1]; however, allowances in the repair design must be made for reductions in mechanical performance arising from the repair application methodology.

### 3.6. Scanning Electron Microscopy

**Figure 12:**
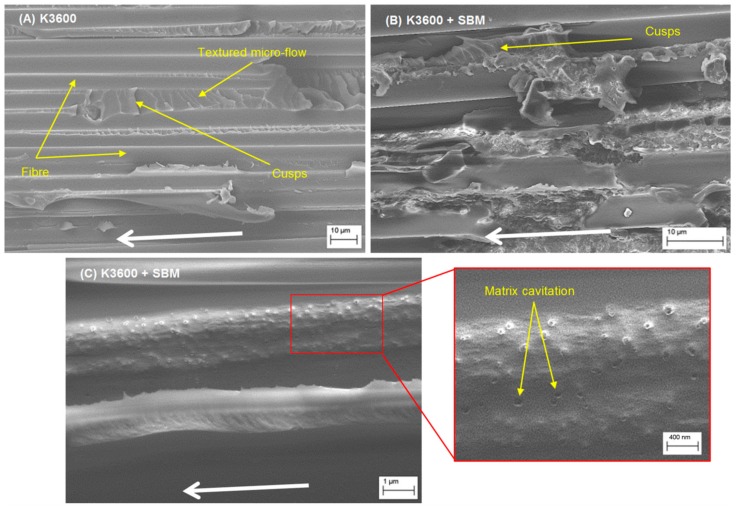
Scanning electron micrographs (SEM) of Neat (**A**) and SBM-toughened (**B**,**C**) fracture surfaces excised from DCB specimens slightly ahead of the final crack tip length. The white arrows show the crack propagation direction.

FESEM results for the autoclave-cured neat and SBM-toughened coupons are shown in Figure 12, with the crack propagation direction in all images running from right to left as indicated by the white arrows. For the neat resin system, Figure 12A, inter-ply resin regions are clearly visible, showing areas of textured micro-flow with angled cusps bridging between adjacent fibres, the angle of which appear to change along the fibre direction. This micro-flow and cusp angle change is likely a result of a changing Mode II fracture component contribution as the crack front propagates along the fibre direction. Also of note is the relative smoothness of the fibres, suggesting poor matrix-to-fibre adhesion in this sample set, which would be a driver for the fibre bridging and pull-out during the testing. In contrast, the SBM-toughened resin system results, Figure 12B,C, appear to be much rougher, suggesting improved fibre to matrix adhesion and a higher proportion of plastic deformation and cohesive failure within the sample set, with this phenomenon also being observed elsewhere in the literature [17]. The inter-fibre matrix regions appear to present less cusping; however, clear evidence of matrix cavitation and nano-structuring of the adhesive can be seen. This cavitation is likely a result of the localised removal of the nano-structures formed by the SBM, which would facilitate void growth and matrix yielding at the crack tip, consequently dissipating local strain energy and leading to improvements in interlaminar toughness. In contrast, for the un-toughened resin system, cohesive fracture within the inter-ply resin regions appears to be the dominant mechanism by which strain energy is dissipated at the crack front. In both neat and toughened resin systems, stray bridging fibres could be seen on the fracture surface; however, they were more prevalent on the un-toughened surface. As discussed previously, these fibres contribute to the apparent material toughness, particularly for longer fracture lengths in the DCB coupon configuration.

**Figure 13:**
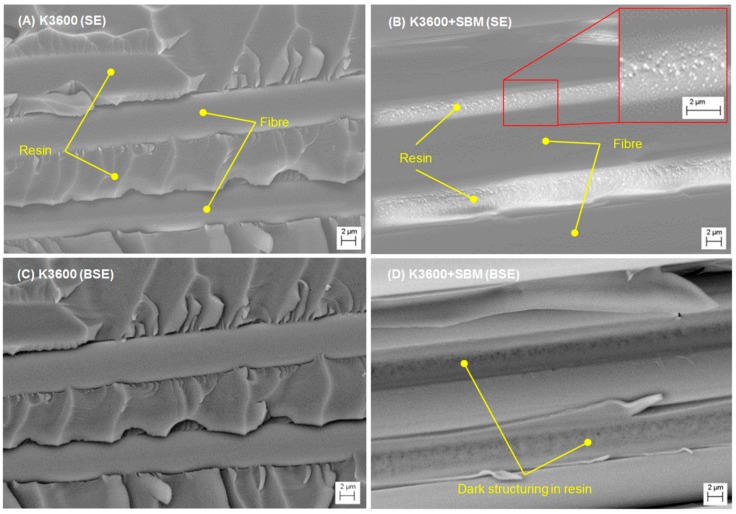
SEM images of Neat (**A**,**C**) and SBM-toughened (**B**,**D**) fracture surfaces excised from the specimens slightly ahead of the final crack tip length. (**A**,**B**) show results from the in-lens secondary electron (SE) detector of the field emission scanning electron microscope (FESEM) while (**C**,**D**) show the results from a backscattered electron (BSE) detector.

To further confirm the formation of nano-structuring by the SBM, comparisons were made between the images from the in-lens secondary electron detector and a backscatter electron detector incorporated into the FESEM imaging column. Backscattered electrons have the advantage that their scattering efficiency is sensitive to the atomic mass of the nuclei they interact with, such that heavier elements will appear brighter due to greater backscattering, while lighter elements will appear darker. Furthermore, backscattered electrons typically have a higher escape depth in the sample, so have less surface resolution, but can provide information on sub-surface structuring. As can be seen from the secondary electron (SE) images in Figure 13A,B, the neat resin presents good resolution of surface features in both samples, with the nano-cavitation of the toughened sample resin regions clearly visible. Comparing this with the respective backscattered electron (BSE) results of Figure 13C,D, little change within the resin regions of the neat sample can be seen (beyond a slight shifting of the image frame); however, the toughened sample presents contrasted structuring within the inter-ply resin regions of the BSE image. This structuring is thought to be due to the immiscible polystyrene block (PS) of the SBM, which would present a different effective atomic number compared to the surrounding epoxy material for the accelerating voltages considered (7–10 kV) and thus a change in the backscattering efficiency.

**Figure 14:**
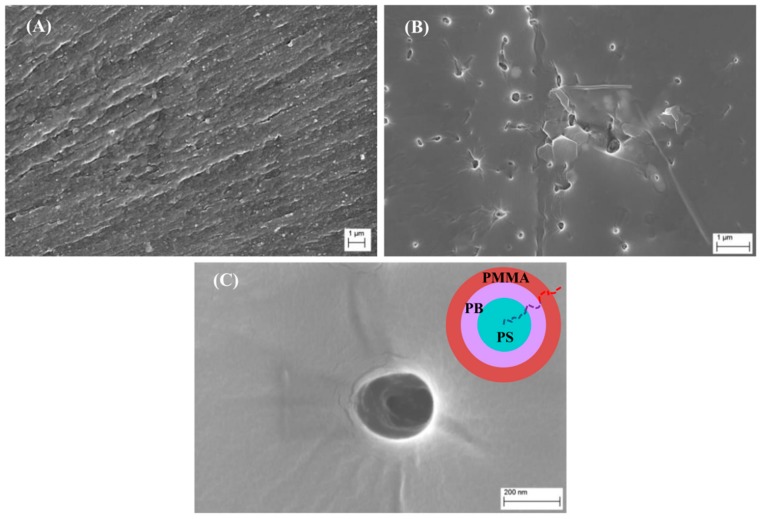
Representative FESEM images of neat (**A**) and toughened K3600 (**B**,**C**) resin samples produced by brittle fracture. The insert in (**C**) shows proposed structuring of the spherical micelle identified in these micrographs and an example copolymer chain from this micelle.

Some representative fracture surfaces from the nitrogen-cooled brittle fracture coupons are shown in Figure 14. As can be seen from these results, the toughened system (Figure 14B,C) presents phase-separated regions on the surface which appear to be spherical micelles. These structures were observed uniformly across the fracture surface and have previously been reported in the literature (e.g., [14,17]). Their formation results from a combination of factors, including [12] solubility of the PMMA block(s) with the epoxy monomer and hardener, complete immiscibility of the PB block within the epoxy monomer, chemical nature of the cross-linker (hardener), chemical composition of the copolymer used (respective molecular weights of each block in the triblock) and concentration of copolymer in the epoxy blend. In Figure 14C, as shown in the insert, the spherical micelles are thought to form with the PMMA block constituting the corona of the micelle, while the immiscible PB and PS blocks form the core of the structure. Removal of these structures from the resin is thought to cause the matrix cavitation observed in the toughened samples of Figure 12. In contrast, the neat sample fracture surface shows no evidence of any surface structuring or phase-separated regions, but rather, regions of brittle fracture.

## 4. Conclusions

An SBM triblock copolymer was successfully used to provide mode I interlaminar fracture toughness improvements in both vacuum bag cured and autoclave cured wet lay-up parts common of typical bonded composite repair applications. Improvements in initiation values of interlaminar fracture toughness were as great as 88% for autoclave-cured parts, with a slight increase in resin *T_g_* and only a small reduction in resin gel time. Thick adherend lap-shear testing yielded a significant improvement in ultimate shear strain of 121% with only an 8% reduction in shear strength. These performance improvements were attributed to the formation of nano-structures within the host matrix, which upon loading, leads to removal of these structures at a crack-tip, resulting in matrix yielding and dissipation of local strain energy. These performance improvements are highly desirable for bonded composite repair applications where fracture toughness and shear performance form critical design requirements for repair resin selection.

## Figures and Tables

**Table 1:** Viscosity measurement results for K3600 and K3600 + E21 (10 wt %) systems.

Temperature	Viscosity	
	K3600	K3600 + E21
°C	Pa·s	Pa·s
15	1.4	16.0
20	0.9	13.8
25	0.6	6.7
30	0.6	6
35	0.2	3.5
